# The relations between temporal and social perceptual biases: Evidence from perceptual matching

**DOI:** 10.3758/s13414-018-01662-8

**Published:** 2019-02-13

**Authors:** Hyunji Kim, Moritz Stolte, Glyn W. Humphreys

**Affiliations:** 1 Department of Psychology, University of Vienna, Vienna, Austria; 2 Department of Experimental Psychology, University of Oxford, Oxford, UK

**Keywords:** Temporal salience, Social salience, Perceptual bias, Decision making bias

## Abstract

We report a new “*now*-bias” effect on simple perceptual matching between shapes and labels and examined the relation between this *now*-bias effect and the *self*-bias previously established with this task (Sui, He, & Humphreys, Journal of Experimental Psychology: Human Perception and Performance, 38, 1105–1117, 2012). The perceptual biases favoring present-relevant and self-relevant information were correlated with each other, suggesting a common underlying mechanism. Nevertheless, temporal biases in decision making, specifically in temporal discounting, correlated with the perceptual *self*-bias but not with the perceptual *now*-bias. We suggest that common attentional biases to present-relevant and self-relevant information mediate perceptual prioritization, whereas temporal discounting is likely involved in a separate reward evaluation mechanism that relates to *self*-bias processes.

**Electronic supplementary material:**

The online version of this article (10.3758/s13414-018-01662-8) contains supplementary material, which is available to authorized users.

Many empirical findings have shown that judgments are often biased toward the self. Self-relevant information is found to be retrieved better than information related to others (e.g., Conway & Pleydell-Pearce, 2000; Symons & Johnson, 1997), and one’s own face is processed faster than other faces (Keyes & Dlgokencka, 2014; Sui & Han, 2007). Most of the previous research demonstrating *self*-biases, however, has been unable to clearly dissociate the observed effects from those of familiarity or exposure. Sui, He, and Humphreys (2012) developed a paradigm that assesses self-biases free from such potential confounds. By using an association approach, the paradigm reveals perceptual prioritization for neutral shapes when they are associated with self-relevant information. Here, we utilized this paradigm to examine perceptual bias in the temporal domain and its relations to *self*-bias (Sui et al., 2012) and to higher-level decision-making (i.e., intertemporal choice).

Accumulating evidence suggests that the conceptualization of the self extends not only to social, but also to temporal, domains (for a review, see Buckner & Carroll, 2007) and that mental representations of such socially and temporally extended selves operate under a shared cognitive mechanism (e.g., Bar-Anan, Liberman, & Trope, 2006; Tamir & Mitchell, 2011; for reviews, see Fiedler, Jung, Wänke, & Alexopoulos, 2012; Liberman & Trope, 2008; Maglio, Trope, & Liberman, 2013). Indeed, the processes of projecting oneself onto a future self and onto another person recruit similar neural networks (e.g., Klein, Loftus, & Kihlstrom, 2002; Mesulam, 2002). Judgments and decisions for socially extended and for temporally extended selves also appear to be analogous (e.g., Pronin & Ross, 2006; Rachlin & Jones, 2008). For instance, decisions made for the future self resemble those made for another person (e.g., Pronin, Olivola, & Kennedy, 2008; Wilson & Gilbert, 2003).

However, to date, evidence showing similar patterns between temporal and social domains at the perceptual level has been missing. The previous research pointing to a common mechanism has largely focused on higher-level decision making involving subjective judgments using self-report measures. One major shortcoming of such measures is that the observed temporal- and self-bias effects may be partially accounted for by effects of familiarity, uncertainty, personal relevance, or importance. By employing the shape-label matching paradigm (Sui et al., 2012), which assigns social (e.g., myself, stranger) or temporal self (e.g., myself right now, myself in 1 year) labels to arbitrary geometric shapes, our aim here was to examine the shared underlying mechanism while eliminating such confounds. We then investigated whether the perceptual prioritization of proximate (i.e., self, now) over distant (i.e., other, later) selves relates to the prioritization of proximate over distant options often observed in higher-level decision-making (i.e., intertemporal choice).

We first examined perceptual matching for temporal self-labels (Exp. 1). We hypothesized that perceptual prioritization for present-relevant stimuli as opposed to future-relevant stimuli would operate in a similar manner as has been shown in the established *self*-bias effect (in which self-relevant information is prioritized over other-relevant information). The results of Experiment 1 supported our hypotheses, establishing a novel *now*-bias effect, in which matching performance is increased for stimuli that are associated with temporal proximity to the self. Subsequently, we assessed whether the perceptual bias in matching temporal labels and shapes (*now*-bias) is related to the perceptual bias in matching social labels and shapes (*self*-bias) at the individual level (Exp. 2). Additionally, in Experiment 2 we evaluated the relations between perceptual biases and a higher-level decision bias, namely temporal discounting (e.g., Green, Fristoe, & Myerson, 1994). Our data suggest that the *now*-biases in perceptual matching and temporal discounting are distinct but that both relate to two mutually exclusive aspects of *self*-bias, most likely attentional modulation and reward evaluation.

## Experiment 1

### Perceptual matching in the temporal domain: A *now*-bias

#### Method

##### Participants

Twenty-five participants (15 females, ten males; *M*_age_ = 28.20 years, *SD*_age_ = 6.75) were recruited at the University of Essex, UK.^1^ Participants received three pounds sterling as compensation. All participants had normal or corrected-to-normal visual acuity.

##### Stimuli and tasks

One of five geometric shapes (hexagon, horizontal ellipse, vertical rectangle, diamond, or cross, each measuring 3.8° × 3.8° of visual angle) was presented above a white fixation cross at the center of the screen. Each shape had previously been associated with one of five temporal self-labels: *Right now*, *Tomorrow*, *In 1 year*, *In 2 years*, and *In 10 years*. The order of presenting the five associations was randomized. Participants subsequently indicated whether the pairings of the shape and label were correct or incorrect, as originally assigned. All stimuli were shown on a gray background on a 24-in. monitor (1,920 × 1,200 pixel resolution at 60 Hz). The experiment was run on a PC using the E-Prime software (version 2.0).

##### Procedure

Participants were instructed to make five associations between shapes and temporal self-labels. For instance, participants read “Diamond is myself in 1 year,” “Hexagon is myself right now,” “Cross is myself in 2 years,” “Horizontal ellipse is myself tomorrow,” “Vertical rectangle is myself in 10 years.” After participants had viewed the shape–label associations and signaled to the experimenter that they had memorized each pair (approximately 1–2 min), participants performed a shape–label matching task. Each trial began with a central fixation cross presented for 500 ms, followed by a pairing of a shape and a label (*Right now*, *Tomorrow*, *In 1 year*, *In 2 years*, *In 10 years*) presented above and below the fixation cross for 100 ms, respectively. The label either matched or did not match the shape as learned. After presenting a shape–label pair, the screen turned blank, allowing up to 1,100 ms for participants to respond. Participants were asked to judge whether the presented shape and label was a correct or an incorrect pairing by pressing keyboard buttons as quickly and accurately as possible. Feedback (*correct*, *incorrect*, or *too slow*) was provided for 500 ms after each trial. Participants completed five blocks of 120 trials each, totaling 600 trials, excluding 20 initial practice trials. Matched and nonmatched combinations occurred equally often in random order. Participants were shown overall accuracy scores at the end of each block.

#### Results and discussion

Following the analyses used in Sui et al. (2012), a 5 (shape category associated with five temporal labels) × 2 (matching judgment: matched vs. nonmatched based on shape) fully repeated measures analysis of variance (ANOVA) on reaction times (RTs) was conducted for correct trials only. Responses shorter than 200 ms were excluded from the analysis (1.5% of all trials). The ANOVA showed a significant effect of shape category, *F*(4, 96) = 2.54, *p* = .045, *η*_p_^2^ = .10 (see Table 1 for the means). Furthermore, we found a significant interaction between shape category and matching judgment, *F*(4, 96) = 4.05, *p* = .004, *η*_p_^2^ = .14.

**Table 1:** Mean reaction times (RTs) and accuracies as a function of matching condition (matching vs. nonmatching) and shape category (*Right now*, *Tomorrow*, *In 1 year*, *In 2 years*, *In 10 years*) in Experiment 1

Conditions	Shape Category	Mean RT (ms)	Accuracy
Matching	Right now	693 (101)	.71 (.23)
	Tomorrow	726 (110)	.62 (.21)
	In 1 year	731 (116)	.59 (.24)
	In 2 years	726 (101)	.61 (.24)
	In 10 years	723 (104)	.66 (.19)
Nonmatching	Right now	751 (121)	.61 (.15)
	Tomorrow	757 (113)	.59 (.18)
	In 1 year	754 (118)	.59 (.16)
	In 2 years	749 (113)	.59 (.18)
	In 10 years	758 (121)	.64 (.17)

RT = reaction time; accuracy = proportion correct. Standard deviations appear within parentheses

Next we conducted analyses for the matched and nonmatched pairs separately. A significant effect of shape category emerged for the matched pairs, *F*(4, 96) = 4.18, *p* = .004, *η*_p_^2^ = .15. After controlling the false discovery rate (FDR; Benjamini & Hochberg, 1995) in multiple pairwise comparisons,^2^ significant differences were found between the *Right now* condition and the *Tomorrow*, *In 1 year*, *In 2 years*, and *In 10 years* conditions (FDR-corrected *p*s = .04, .02, .02, .05, respectively; other *p*s > .82). A significant linearity indicating faster RTs for the *Right now* condition emerged, *F*(1, 24) = 6.33, *p* = .02. The nonmatched pairs showed no significant effects of shape category (*p*s > .68). Accuracy scores showed a similar pattern of results (see the supplemental material).

The results confirmed that in a simple shape-matching paradigm using arbitrary geometric shapes associated with temporal self-labels, systematic biases emerge in which stimuli associated with the present self are matched more rapidly than those associated with future selves. An overall linear relationship emerged as a function of temporal label, although the greatest shift was between now and all future times. Thus, our results provide the first demonstration of temporal information modulating simple perceptual matching.

An alternative explanation of the results might be that participants memorized the shape and label pairs in chronological order, and thus, the shapes associated with more distant temporal labels might have taken longer to retrieve. If this were true, we would have observed the same RT increase for the temporally distant shapes in the nonmatching pairs. However, this was not the case.

In Experiment 2, we compared the *now*-bias observed in Experiment 1 with (i) the *self*-bias found for the processing of social labels and (ii) the *present*-bias commonly observed in intertemporal choice tasks (i.e., temporal discounting). According to previous findings, the cognitive biases affecting temporal and social labels may share a common mechanism (e.g., Bar-Anan et al., 2006). Thus, we hypothesized that (i) current prioritization in perception and temporal discounting should be correlated with *self*-bias, and (ii) if biases in low- and high-level processes overlapped, temporal biases in perceptual matching and intertemporal choice should also correlate with each other. We tested these hypotheses in Experiment 2.

## Experiment 2

### Relations between *self*- and *now*-biases in perceptual matching and temporal discounting

Using a within-participants design, we employed an intertemporal choice survey followed by a lab experiment involving two shape–label matching tasks assessing effects of the *now*-bias and the *self*-bias on perceptual matching.

#### Method

##### Participants

Forty participants (25 females, 15 males; *M*_age_ = 26.53 years, *SD*_age_ = 5.90) from the University of Essex took part in exchange for five pounds sterling. Participants filled out an online intertemporal choice survey, and only those who completed the survey took part in the lab experiment.

##### Intertemporal choice

To measure temporal discounting, we tested participants using an adaptive procedure modeled on the estimation of psychophysical thresholds (e.g., Ungemach, Stewart, & Reimers, 2011). In this particular case, we measured participants’ discounted values of receiving £1,000 one year from the present time. Across a number of choice-making questions, participants chose between receiving a sooner, smaller reward or a later, bigger reward. Depending on the individual choices, the amount of money offered for the *now* option increased or decreased systematically on the next question. When participants switched their choices, either from the *now* to the *later* option or from the *later* to the *now* option, the average of the two values offered for the *now* options in the previous and current trials formed the indifference point. The indifference point indicates a participant’s current discounted value of £1,000 one year from now, with higher indifference points indicating weaker temporal discounting (more patient responses).

##### Stimuli and tasks

For each participant, three out of six geometric shapes (hexagon, horizontal ellipse, vertical rectangle, diamond, cross, and reversed triangle, each measuring 3.8° × 3.8°) were randomly assigned to three temporal conditions (*Right now*, *Tomorrow*, *In 1 year*). The remaining shapes were assigned to three social conditions (self, friend, stranger). The rest of the stimuli and tasks remained identical to those in Experiment 1.

##### Procedure

Participants completed the online survey, followed by the laboratory experiment involving two tasks: temporal self and social shape–label matching tasks. The order of the two tasks was counterbalanced across participants. Participants were instructed to associate three shapes with three labels (i.e., [myself] *Right now*, *Tomorrow*, *In 1 year* in the temporal-self label task, or *Self, Friend, Stranger* in the social label task) prior to each of the two matching tasks. For each task, participants completed three blocks of 120 trials, excluding 12 initial practice trials. The rest of the procedure remained identical to that of Experiment 1.

#### Results

##### Now-bias and self-bias: Effects of shape category (ANOVAs)

Two separate 3 (three shapes associated with temporal or social labels) × 2 (matching judgment: matching vs. nonmatching) ANOVAs were conducted on correct RTs (see Table 2 for the means). Responses shorter than 200 ms were excluded (1.4% of all trials). The assumption of sphericity was violated for the main effects of shape category for both the temporal, *χ*^2^(2) = 10.45, *p* = .005, and social, *χ*^2^(2) = 6.74, *p* = .03, conditions. Therefore, degrees of freedom were corrected using Greenhouse–Geisser estimates of sphericity (*ε* = .81 for the temporal and *ε* = .86 for social conditions).

**Table 2:** Mean reaction times (RTs) and accuracies as a function of matching condition (matching vs. nonmatching) and shape category in Experiment 2

Task	Conditions	Shape Category	Mean RT (ms)	Accuracy
Social labels	Matching	Self	656 (68)	.90 (.12)
		Friend	719 (81)	.82 (.16)
		Stranger	742 (81)	.65 (.21)
	Nonmatching	Self	756 (79)	.77 (.16)
		Friend	761 (81)	.76 (.18)
		Stranger	763 (83)	.78 (.18)
Temporal labels	Matching	Right now	687 (91)	.85 (.14)
		Tomorrow	720 (98)	.78 (.17)
		In 1 year	717 (101)	.79 (.15)
	Nonmatching	Right now	750 (100)	.74 (.16)
		Tomorrow	762 (104)	.75 (.16)
		In 1 year	756 (105)	.75 (.17)

RT = reaction time; accuracy = proportion correct. Standard deviations appear within parentheses

In the temporal matching task, a reliable main effect of shape category was revealed, *F*(1.61, 62.88) = 8.45, *p* = .001, *η*_p_^2^ = .18. A significant interaction between shape category and matching judgment was also observed, *F*(2, 78) = 5.17, *p* = .008, *η*_p_^2^ = .12. Next, analyses for the matched and nonmatched pairs were conducted separately. The degrees of freedom were corrected using the Greenhouse–Geisser estimate of sphericity (*ε* = .85), due to violation of sphericity, *χ*^2^(2) = 7.43, *p* = .02. A significant effect of shape category for the matched pairs was observed, *F*(1.70, 66.24) = 9.32, *p* = .001, *η*_p_^2^ = .19. FDR-controlled pairwise comparisons revealed significantly faster responses in the *Right now* than in the *Tomorrow* and *In 1 year* conditions (both FDR-corrected *p*s = .01). The test of linearity also reached significance, *F*(1, 39) = 9.88, *p* = .003. The effect for nonmatched pairs was marginal, *F*(2, 78) = 2.57, *p* = .08, *η*_p_^2^ = .06, and no significant differences were found in nonmatched pairs (all *p*s > .16). Largely similar results were obtained for accuracy scores (see the supplemental material).

In the social matching task, a reliable main effect of shape category was observed, *F*(1.72, 67.10) = 31.86, *p* < .001, *η*_p_^2^ = .45, as well as a significant interaction between shape category and matching judgment, *F*(2, 78) = 37.60, *p* < .001, *η*_p_^2^ = .49. We also observed a significant effect of shape category in the matched pairs, *F*(2, 78) = 43.73, *p* < .001, *η*_p_^2^ = .53. FDR-controlled comparisons revealed significant differences between all conditions (self–friend, *p* = .01, self–stranger, *p* = .01, friend–stranger, *p* = .02). The test of the linear effect of shape category was also significant, *F*(1, 39) = 65.10, *p* < .001. Nonmatched pairs showed no significant effects of shape category (all *p*s > .38). Accuracy scores revealed a similar pattern of results (see the supplemental material).

##### Correlations

To test the relationship between perceptual biases and the effects of temporal discounting in the intertemporal choice task, we computed RT and accuracy differences between all conditions within each temporal and social matching task (e.g., RTs for tomorrow **–** RTs for right now; RTs for stranger **–** RTs for self; etc.).

**Fig. 1:**
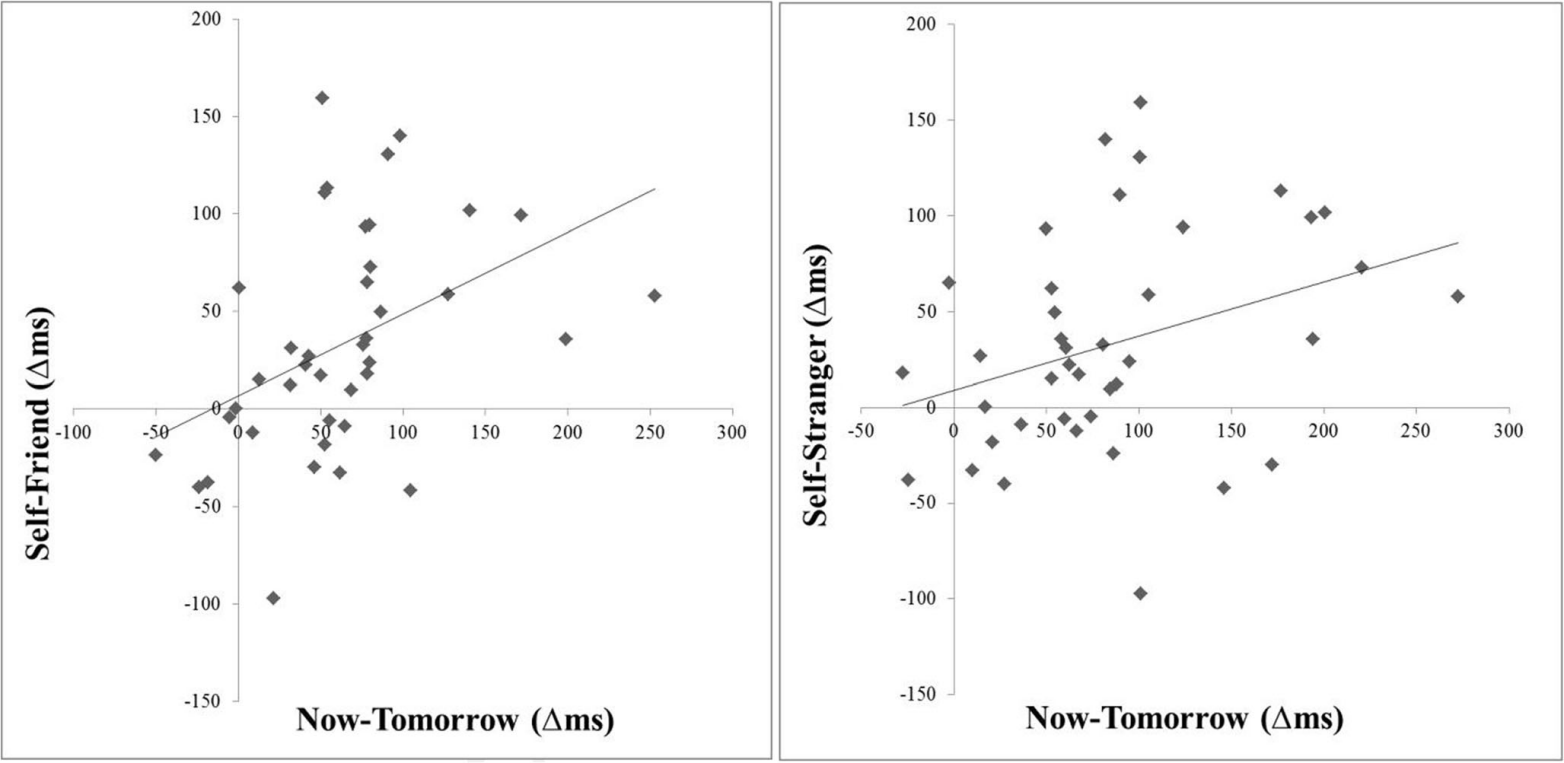
Intercorrelations in reaction times between the *now*-bias (vs. tomorrow) and the *self*-bias (vs. friend or stranger) in the shape–label matching task in Experiment 2

For the RT data, significant correlations were revealed between the now–tomorrow contrast and the self-biases in the self–friend contrast, *r*(38) = .43, *p* = .006, and the self–stranger contrast, *r*(38) = .34, *p* = .035: The more *self*-bias participants exhibited, the more *now*-bias they also showed (see Fig. 1). The other intercorrelations were not significant (e.g., between the *friend*-bias [friend–stranger difference] and the now-bias, all *p*s > .12; see Table S1 in the supplemental material for the full intercorrelations).

**Fig. 2:**
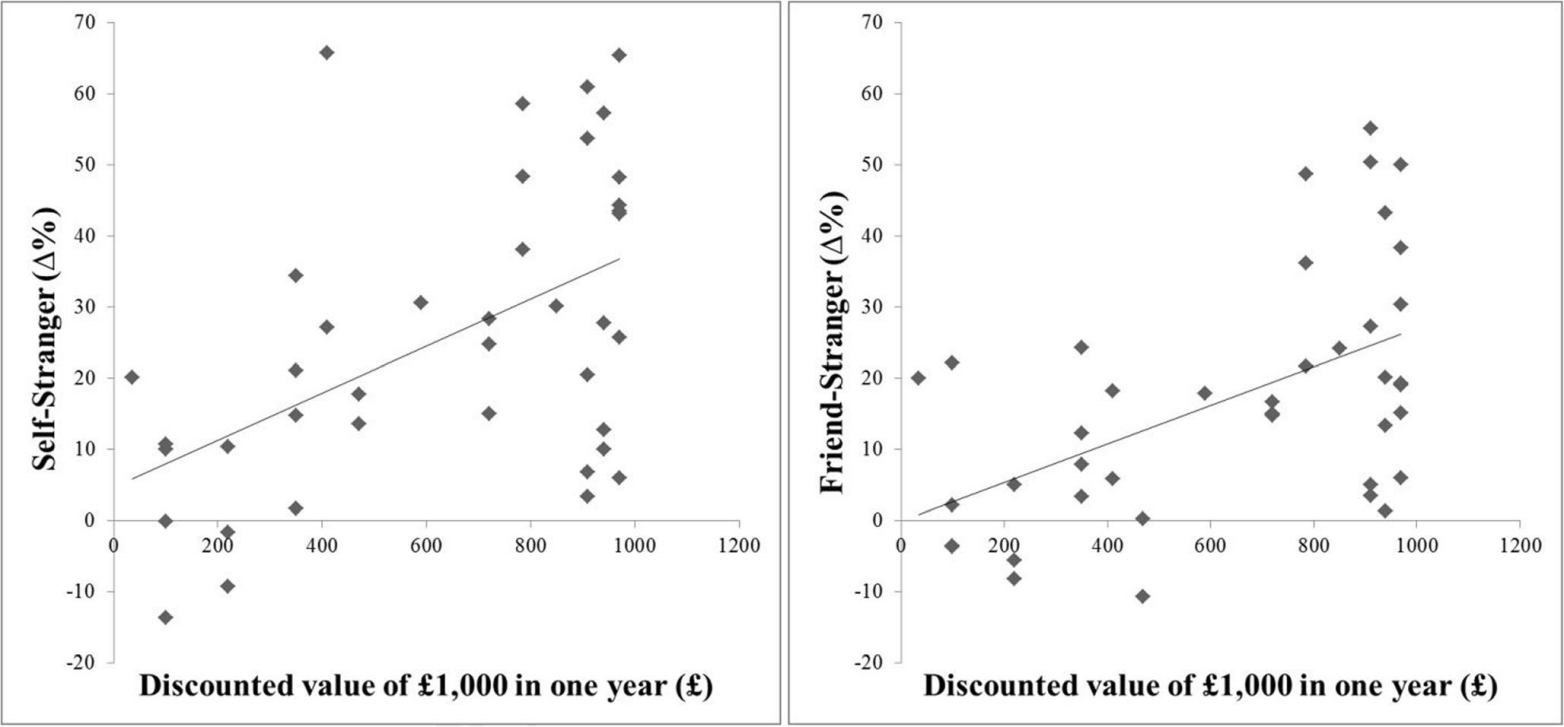
Intercorrelations of the *self*-bias and the *friend*-bias in accuracy with temporal discounting in Experiment 2. “Discounted value of £1,000 in one year” indicates individual indifference points assessed in the intertemporal choice survey

For accuracy scores, significant positive correlations were observed between the self–stranger contrast (*self*-bias) and temporal discounting (*M* = 632.75, *SD* = 327.19), *r*(38) = .52, *p* = .001, and between the friend–stranger contrast (*friend*-bias) and temporal discounting, *r*(38) = .53, *p* = .001, indicating that the stronger the *self-* or *friend*-bias participants had, the less temporal discounting they exhibited (see Fig. 2). In other words, when participants had a stronger bias toward the more familiar target (i.e., self, friend), they made more patient choices on the intertemporal choice survey. Interestingly, there were no significant correlations between the *now*-bias in the perceptual matching task and temporal discounting (all *p*s > .38). We discuss a possible explanation for these correlations in the General Discussion.

#### Discussion

In Experiment 2, we replicated the *now*-bias observed in Experiment 1. As hypothesized, the *now*-bias related to the *self*-bias effect, supporting our hypothesis of a shared mechanism.

The biases to the *friend* and the *self* in perceptual matching both correlated with temporal discounting (i.e., the stronger the bias, the weaker the discounting), suggesting that the more sensitive a participant was to the closeness of a social target, the more the participant was willing to stave off discounting. This contrasts with the more specific correlation between the *self-*bias (but not the *friend-*bias) and the *now-*bias. Furthermore, the perceptual *now*-bias did not correlate with the temporal-discounting effect. The latter results indicate a distinction between, on the one hand, specific *self-* and *now-*biases on perceptual matching and, on the other, a *self*- and *friend*-bias, and temporal discounting. We review this distinction in the General Discussion.

Finally, we explored an alternative account for the prioritization effect, suggesting that the behavioral patterns observed in Experiments 1 and 2 might have been due to a memory order effect rather than to a perceptual effect. For instance, when learning a set of social or temporal information, participants might form a mental list of information in a social or temporal order and use this memory list when performing the shape-matching task, thus prioritizing the first item on the list (i.e., self or right now). The memory order account argues that what underlies the perceptual prioritization effect might not be the increased perceptual saliency of specific social information, but rather the order of how social information is stored in memory. Although in the learning phase we presented the labels in a random order, our results did not rule out this alternative explanation. To resolve this problem, we designed Experiment 3 to test the memory order account.

## Experiment 3

### Test of memory order effects in perceptual matching

If the memory order account was responsible for the prioritization effect, a set of social information that could easily be ordered to anyone should also show the bias effect we had observed in Experiments 1 and 2. To test this, we chose control labels that reflected a systematic increase in quantity, and thus had an obvious order, but did not involve any dimension of psychological distance (e.g., spatial, numeric increase, social, or temporal). As control labels, we chose *myself after drinking a*: – “sip” of water, – “glass” of water, – “bottle” of water, and tested whether participants would show a *sip*-bias.

Using a within-participants design, we conducted a lab experiment involving two shape–label matching tasks assessing the effects of *self*-bias and a possible *sip*-bias on perceptual matching.

#### Method

##### Participants

Thirty-eight participants (30 females, eight males; *M*_age_ = 21.58 years, *SD*_age_ = 3.48) from the University of Vienna took part in exchange for course credits.

##### Stimuli and tasks

For each participant, three out of six geometric shapes (hexagon, horizontal ellipse, vertical rectangle, diamond, cross, and reversed triangle, each measuring 3.8° × 3.8°) were randomly assigned to three social conditions (self, friend, stranger) and three control conditions (sip, glass, bottle). The rest of the stimuli and tasks remained identical to the matching tasks used in Experiment 2, except that the instruction and the labels were translated into German.

##### Procedure

Participants performed two matching tasks in a counterbalanced order across participants. The rest of the procedure remained identical to that of the matching tasks used in Experiment 2.

#### Results

##### Self-bias and sip-bias: Effects of shape category (ANOVAs)

Two separate 3 (three shapes associated with social and control labels) × 2 (matching judgment: matching vs. nonmatching) ANOVAs were conducted on correct RTs (see Table 3 for the RT and accuracy means). Responses shorter than 200 ms were excluded (5.3% of all trials).

**Table 3:** Mean reaction times (RTs) and accuracies as a function of matching condition (matching vs. nonmatching) and shape category in Experiment 3

Task	Conditions	Shape Category	Mean RT (ms)	Accuracy
Control labels	Matching	Sip	659 (56)	.85 (.08)
		Glass	645 (58)	.86 (.12)
		Bottle	649 (61)	.87 (.13)
	Nonmatching	Sip	705 (51)	.89 (.08)
		Glass	718 (52)	.86 (.10)
		Bottle	713 (56)	.87 (.10)
Social labels	Matching	Self	605 (52)	.95 (.04)
		Friend	674 (52)	.88 (.07)
		Stranger	691 (53)	.81 (.11)
	Nonmatching	Self	718 (53)	.89 (.09)
		Friend	733 (55)	.83 (.12)
		Stranger	726 (48)	.86 (.10)

RT = reaction time; accuracy = proportion correct. Standard deviations appear within parentheses

The assumption of sphericity was violated for the main effects of shape category in the control labels, *χ*^2^(2) = 6.00, *p* = .05, and marginally for the social labels, *χ*^2^(2) = 5.67, *p* = .06. Therefore, degrees of freedom were corrected using Greenhouse–Geisser estimates of sphericity (*ε* = .87 for the control and *ε* = .87 for social labels).

In the control task, a main effect of shape category was not observed, *F*(1.73, 64.15) = 0.05, *p* = .94, but an interaction between shape category and matching judgment, *F*(1.95, 72.05) = 4.40, *p* = .02, indicated that participants responded to “glass”-associated shapes faster when they were matching but slower when they were mismatching, as compared to the “sip”- and “bottle”-associated shapes. However, the nonsignificant main effect indicated that even when given a clear order (i.e., increasing in quantity), the perceptual prioritization does not necessarily occur, thus ruling out the memory order account. Meanwhile, the same participants showed a significant effect of shape category in the social matching task, *F*(1.75, 64.59) = 54.72, *p* < .001, *η*_p_^2^ = .60, with a significant interaction between shape category and matching judgment, *F*(2, 73.96) = 62.58, *p* < .001, *η*_p_^2^ = .63, replicating the prioritization pattern observed in Experiment 2.

#### Discussion

Experiment 3 tested an alternative explanation, that perceptual prioritization might have been due to an memory order effect, but our results revealed that using labels that increased in a specific order did not suffice for the perceptual-matching effect to arise. It is important to note that in the control matching task, we instructed participants to relate the control labels to the self by giving them instructions to make associations between the shapes and themselves after drinking a sip, glass, or bottle of water; a very similar design to the temporal matching task used in Experiment 2. Taken together, our results suggest that the perceptual prioritization effect may not always occur when a set of labels can form any self-relevant order, but only when the labels form a self-relevant order as a function of social or temporal dimension.

## General discussion

We first demonstrated a *now*-bias in processing of temporal self information in a simple perceptual-matching task, providing novel evidence that temporal self-biases modulate perceptual matching. Interestingly, we demonstrated that the two biases in perceptual matching are related at the individual level: Participants showing a large *self*-bias also had a large *now*-bias. Our data are consistent with prior suggestions that the two biases might share a common underlying mechanism (e.g., Buckner & Carroll, 2007). The present findings also extend previous evidence of the shared-mechanism account in higher-level decision making (e.g., Wilson & Gilbert, 2003) to the perceptual level, while ruling out confounds such as familiarity, uncertainty, personal relevance, and importance.

Interestingly, temporal discounting observed in intertemporal choice did not correlate with the *now*-bias, but it did correlate with the *self*- and *friend*-biases. These results suggest that the *now*-bias and temporal discounting might tap into two different mechanisms that are mutually exclusive: attentional modulation and reward evaluation, both of which have been shown to be related to *self*-bias. Salient social information is prioritized by attention, as has been demonstrated for self-relevant as compared to other-relevant information (Humphreys & Sui, 2016; Sui, Liu, Mevorach, & Humphreys, 2015). Our findings suggest that present-relevant information might be prioritized in a similar way. Reward evaluation, on the other hand, is involved when making intertemporal choices in decision making, requiring people to weigh the values of present and future rewards (Loewenstein & Prelec, 1992). Reward has also previously been implicated as a factor explaining the observed self-prioritization in perceptual matching (Sui et al., 2012). Sui and Humphreys (2015a, 2015b) reported evidence that there are both common and distinct effects of reward and *self*-bias. For example, using more than one self-associated stimulus increased *self*-bias effects, whereas using more than one reward-associated stimulus did not increase reward effects (Sui & Humphreys, 2015b). Nevertheless, individuals who rated the personal distance between themselves and others as large were minimally affected by variations in reward linked to self and to other stimuli, whereas individuals who reported a close distance between themselves and others were more affected by having different reward values associated with the self and with others (Sui & Humphreys, 2015a). Thus, we argue that the *self*- and *friend*-biases may reflect reward-related aspects of decision making, and therefore correlate with temporal discounting across individuals.

Overall, our findings support a common mechanism for perceptual prioritization of self-relevant information in the social and temporal dimensions. However, although perceptual *self*-bias predicts temporal discounting, presumably, in the case of reward processing, the perceptual *now*-bias seems to bear no relationship to temporal discounting. We suggest that future research should examine such converging and diverging aspects of processes between perceptual biases and higher-level decision biases in social information processing, to clarify the extent to which higher-level decision biases involving social and temporal dimensions permeate early-level biases.

### Author note

This work was supported by grants from a European Research Council Advanced Investigator award (Pepe: 323883), the Economic and Social Research Council (UK, ES/J001597/1), and a Wellcome Trust Senior Investigator award (WT 106164MA) to the last author.

## Electronic supplementary material

ESM 1 (DOC 42 kb)

## Footnotes

1 Our sample sizes were fixed ahead of data collection in order to obtain power greater than 80% to detect a moderate effect size (i.e., *f* = .25, *α* = .05).

2 In contrast to family-wise techniques of control such as the Bonferroni correction, allowing low likelihood of detecting effects (leaving researchers more exposed to Type II error; Keselman, Cribbie, & Holland, 2002), controlling the FDR introduces a better compromise between Type I and Type II errors by allowing an adaptive criterion when performing multiple comparisons. Thus, to increase power, the FDR procedure can be preferred to family-wise techniques (Benjamini & Hochberg, 1995). The procedure in the present study followed the steps provided at https://www.marum.de/Binaries/Binary745/BenjaminiHochberg.xlsx.
