# Supplementary material for: The relations between temporal and social perceptual biases: Evidence from perceptual matching
Source: Atten Percept Psychophys. 2019 Feb 13;81(3):599–606. doi: 10.3758/s13414-018-01662-8 (PMC6407911; doi:10.3758/s13414-018-01662-8)
Supplement: Supplementary file 1 — (DOC 42 kb) [file 13414_2018_1662_MOESM1_ESM.doc]

**Supplemental Material**

**Experiment 1: Analysis of accuracy data**

An ANOVA on Accuracy (ACC) for correct trials revealed a significant effect of shape category, *F*(2.95, 70.88) = 4.28, *p* = .008, *η_p_*^2^ = .15 (the degrees of freedom corrected using the Greenhouse-Geisser estimate of sphericity (ε = .74), due to violation of sphericity, χ^2^(9) = 20.04, *p* = .02; see Table 1 for means). The interaction was not significant, *F*(2.60, 62.54) = 1.61, *p* = .20. The analysis for matched pairs revealed a significant effect of shape category (Greenhouse-Geisser corrected; ε = .67; χ^2^(9)= 23.43, *p* = .005), *F*(2.66, 63.83) = 3.16, *p* = .04, *η_p_*^2^ = .12. Pairwise comparisons revealed marginally significant differences between the *Right now* condition and the *Tomorrow*, *In 1 year*, and *In 2 years* conditions (FDR corrected *p*s = .06, .06, .06, respectively, other *p*s > .11).

**Experiment 2 – Temporal label matching task: Analysis of accuracy data**

A significant effect of shape category on ACC, *F*(2, 78) = 4.87, *p* = .01, *η_p_*^2^ = .11., and a significant interaction with matching judgement, *F*(1.72, 66.95) = 3.64, *p* = .04, *η_p_*^2^ = .09 (Greenhouse-Geisser corrected, ε = 86; χ^2^(2) = 6.85, *p* = .03) were revealed. A significant effect of shape category was observed in matched pairs, *p* = .003 but not in non-matched pairs, *p* = .89. FDR controlled comparisons for matched pairs showed significantly higher ACCs in the ‘Right now’ condition compared to ACCs in the ‘Tomorrow’ condition, corrected *p* = .01 (other *p*s > .22).

**Experiment 2 – Social label matching task: Analysis of accuracy data**

Analyses of ACC showed a reliable effect of shape category, *F*(2, 78) = 34.99, *p* < .001, *η_p_*^2^ = 47, and an interaction with matching judgement, *F*(2, 78) = 28.53, *p* < .001, *η_p_*^2^ = .42. A significant effect of shape category was observed in matched pairs, *p* < .001, showing significant differences between all possible pairs (FDR corrected *p*s = .01), but not in non-matched pairs, *p*s > .67.

**Table 1S*.* Reaction times (RTs) and accuracy (ACC) Inter-correlations in Experiment 2.**

|  | 1. | 2. | 3. | 4. | 5. | 6. | 7. |
| --- | --- | --- | --- | --- | --- | --- | --- |
| 1. Self-Friend | -- | .60** | -.12 | .21 | .12 | -.05 | .13 |
| 2. Self-Stranger | .63** | -- | .73** | .22 | .23 | .05 | .52** |
| 3. Friend-Stranger | -.29 | .56** | -- | .10 | .18 | .11 | .53** |
| 4. Now-Tomorrow | .43** | .34* | -.05 | -- | .48** | -.36 | .06 |
| 5. Now-One year | .25 | .20 | -.02 | .76** | -- | .65 | .14 |
| 6. Tomorrow-One year | -.22 | -.16 | .04 | -.25 | .45** | -- | .10 |
| 7. Indifference Point | .02 | .20 | .23 | -.004 | .05 | .07 | -- |

Note: The bottom left panel indicates correlation coefficients for the RT data and the top right panel indicates correlation coefficients for the ACC data in Experiment 2.

*p* < .05 = *, *p* < .01 = **
